# Hypoinsulinemia Regulates Amphetamine-Induced Reverse Transport of Dopamine

**DOI:** 10.1371/journal.pbio.0050274

**Published:** 2007-10-16

**Authors:** Jason M Williams, W. Anthony Owens, Gregory H Turner, Christine Saunders, Concetta Dipace, Randy D Blakely, Charles P France, John C Gore, Lynette C Daws, Malcolm J Avison, Aurelio Galli

**Affiliations:** 1 Department of Molecular Physiology and Biophysics, Vanderbilt University Medical Center, Nashville, Tennessee, United States of America; 2 Vanderbilt University Institute of Imaging Science, Vanderbilt University Medical Center, Nashville, Tennessee, United States of America; 3 Center for Molecular Neuroscience, Vanderbilt University Medical Center, Nashville, Tennessee, United States of America; 4 Department of Physiology, The University of Texas Health Science Center, San Antonio, Texas, United States of America; 5 Department of Radiology and Radiological Sciences, Vanderbilt University Medical Center, Nashville, Tennessee, United States of America; 6 Department of Pharmacology, Vanderbilt University Medical Center, Nashville, Tennessee, United States of America; 7 Department of Pharmacology, The University of Texas Health Science Center, San Antonio, Texas, United States of America; 8 Department of Psychiatry, The University of Texas Health Science Center, San Antonio, Texas, United States of America; University of Texas Southwestern Medical Center, United States of America

## Abstract

The behavioral effects of psychomotor stimulants such as amphetamine (AMPH) arise from their ability to elicit increases in extracellular dopamine (DA). These AMPH-induced increases are achieved by DA transporter (DAT)-mediated transmitter efflux. Recently, we have shown that AMPH self-administration is reduced in rats that have been depleted of insulin with the diabetogenic agent streptozotocin (STZ). In vitro studies suggest that hypoinsulinemia may regulate the actions of AMPH by inhibiting the insulin downstream effectors phosphotidylinositol 3-kinase (PI3K) and protein kinase B (PKB, or Akt), which we have previously shown are able to fine-tune DAT cell-surface expression. Here, we demonstrate that striatal Akt function, as well as DAT cell-surface expression, are significantly reduced by STZ. In addition, our data show that the release of DA, determined by high-speed chronoamperometry (HSCA) in the striatum, in response to AMPH, is severely impaired in these insulin-deficient rats. Importantly, selective inhibition of PI3K with LY294002 within the striatum results in a profound reduction in the subsequent potential for AMPH to evoke DA efflux. Consistent with our biochemical and in vivo electrochemical data, findings from functional magnetic resonance imaging experiments reveal that the ability of AMPH to elicit positive blood oxygen level–dependent signal changes in the striatum is significantly blunted in STZ-treated rats. Finally, local infusion of insulin into the striatum of STZ-treated animals significantly recovers the ability of AMPH to stimulate DA release as measured by high-speed chronoamperometry. The present studies establish that PI3K signaling regulates the neurochemical actions of AMPH-like psychomotor stimulants. These data suggest that insulin signaling pathways may represent a novel mechanism for regulating DA transmission, one which may be targeted for the treatment of AMPH abuse and potentially other dopaminergic disorders.

## Author Summary

Abuse of psychostimulants such as amphetamine remains a serious public health concern. Amphetamines mediate their behavioral effects by stimulating dopaminergic signaling throughout reward circuits of the brain. This property of amphetamine relies on its actions at the dopamine transporter (DAT), a presynaptic plasma membrane protein that is responsible for the reuptake of extracellular dopamine. Recently, we and others have revealed the novel ability of insulin signaling pathways in the brain to regulate DAT function as well as the cellular and behavioral actions of amphetamine. Here we used a model of Type I diabetes in rats to uncover how insulin signaling regulates DAT-mediated amphetamine effects. We show that by depleting insulin, or through selective inhibition of insulin signaling, we can severely attenuate amphetamine-induced dopamine release and impair DAT function. Our findings demonstrate in vivo the novel ability of insulin signaling to dynamically influence the neuronal effects of amphetamine-like psychostimulants. Therefore, the insulin signaling pathway, through its unique regulation of brain dopamine, may be targeted for the treatment of amphetamine abuse.

## Abstract 3

After the striatum is exposed to amphetamines, insulin signaling regulates dopamine via downstream phosphotidylinositol 3-kinase and protein kinase B targets. This in vivo evidence points toward a mechanism for drug abuse.

## Introduction

Virtually all major classes of abused drugs share an ability to enhance dopamine (DA) transmission throughout midbrain reward centers [1,2]. Once DA is released into the synapse, the DA transporter (DAT) is the primary mechanism for clearing the transmitter from the extracellular space, particularly within the striatum [3–5]. DAT is a target of multiple psychomotor stimulants including cocaine, methamphetamine and amphetamine (AMPH) [6]. Dysregulation of DAT function has been implicated in a wide variety of neuropsychiatric pathologies, including attention-deficit hyperactivity disorder, depression and bipolar disorder [1,7].

DA clearance is dynamically modulated by several signaling pathways [8–10]. Importantly, recent studies suggest a unique role for insulin and insulin-like growth factors (e.g., IGF1 and IGF2) in this modulation [11–14]. Insulin receptors (IRs) and receptors for IGF1–2 are found on DAT-expressing midbrain DA neurons [15–18]. Insulin and IGF1–2 receptors function as receptor tyrosine kinases (RTKs), which have been shown to regulate the activity of a variety of neurotransmitter transporters [19–22]. Additionally, RTKs are known to stimulate phosphotidylinositol 3-kinase (PI3K) signaling, which in turn activates protein kinase B (PKB), also known as Akt [23,24]. Akt is a central player in insulin and growth factor signaling and a regulator of several cellular functions including cell growth and apoptosis [25]. Recently, the PI3K/Akt signaling pathway has been shown to regulate DA clearance [11] and has been implicated in cocaine sensitization [26], alcohol tolerance [27] and opioid dependence [28]. The mechanism underlying the regulation of DA clearance by PI3K seems to rely on DAT trafficking, as Garcia et al. [13] and Wei et al. [29] recently demonstrated that Akt activity is critical for sustaining human DAT (hDAT) membrane expression and function.

In vivo evidence supporting insulin and PI3K signaling pathways in the control of DA clearance comes from Patterson et al. [30], who demonstrated that in rats, hypoinsulinemia induced by food deprivation decreases the maximum velocity [*V*_max_] for DA uptake (with no significant change in the affinity constant [*K*_m_] for DA), as determined by rotating disk voltammetry on striatal suspensions. Consistently, the uptake of DA, as determined ex vivo by using striatal synaptosomes and in vivo by high-speed chronoamperometry (HSCA), is severely reduced in rats previously depleted of insulin with the diabetogenic agent streptozotocin (STZ) [14].

AMPH-like stimulants are actively transported by catecholamine carriers such as DAT [6]. As substrates, AMPHs not only competitively inhibit DA reuptake and thereby increase synaptic DA, but also promote reversal of transport, resulting in efflux of DA via the DAT [6]. This efflux results in an increase in extracellular DA and is believed to be of major importance for the psychomotor stimulant properties of AMPHs [6]. Because insulin and PI3K signaling have been shown to fine-tune DAT cell surface expression [13,29], it is possible that inhibition of PI3K signaling in vivo*,* by reducing DAT cell surface expression, inhibits AMPH-induced DA efflux and, hence, its behavioral effects. The ablation of pancreatic β cells by STZ in rats is a model of insulin depletion, and as such, we hypothesized that PI3K signaling in the brain, as well as DAT cell surface expression and possibly DAT-mediated behavioral effects of AMPH, would be reduced following STZ pretreatment. In support of our hypothesis are studies showing that, insulin-depleted, diabetic rodents have significantly reduced basal locomotor activity [14,31,32] and are resistant to the motor stimulant properties of AMPH and other related psychomotor stimulants [31,33,34]. Likewise, the reinforcing potential of AMPH, as determined by the daily maintenance of intravenous AMPH self-administration, is significantly blunted in the STZ model of hypoinsulinemia [12]. Therefore, in light of these data, it is possible that the regulation of AMPH-induced DA efflux—promoted by insulin and PI3K signaling—is mediated by changes in DAT cell surface expression.

Here we show that pharmacological manipulation of the PI3K signaling pathway caused by hypoinsulinemic conditions or selective pharmacological inhibition/activation of PI3K dramatically regulates the ability of AMPH to evoke DAT-mediated DA release in the striatum, as determined by HSCA. Consistently, in hypoinsulinemic rats we observed a blunting of AMPH-evoked striatal activation measured by functional magnetic resonance imaging (fMRI). We couple these findings with biochemical data showing that PI3K/Akt signaling is reduced under hypoinsulinemic conditions, as is the cell surface distribution of the DAT within striatum. Collectively, these data support the novel concept that insulin signaling—possibly through PI3K and/or Akt—plays a critical role in DA homeostasis by regulating DA clearance and the increases in extracellular DA induced by AMPH-like psychomotor stimulants.

## Results

### STZ Markedly Reduces AMPH-Induced DA Release in Striatum

PI3K signaling, which is stimulated by activation of IRs and other RTKs [23], plays a critical role in the maintenance of DA clearance and DAT cell surface expression [11,13,21,35]. Therefore, it is conceivable that PI3K signaling and ultimately Akt, by fine-tuning DAT expression at the plasma membrane [13,29], regulate the ability of AMPH to cause DAT-mediated DA efflux.

To test this hypothesis, we first altered PI3K signaling in vivo by depleting circulating plasma levels of insulin, a potent hormonal activator of the PI3K/Akt pathway [23,24], using the antibiotic STZ [36]. We administered a single dose of STZ (65 mg/kg) by tail vein injection at least 7 d prior to experiments. This regimen led to a significant increase in blood glucose levels: 532 ± 39 mg/dl (STZ-treated rats) versus 108 ± 21 mg/dl (untreated controls) (*p* < 0.001, Students *t*-test; *n* = 11–12 rats). Radioimmunoassay data from our laboratory suggest that STZ reduces striatal levels of insulin by at least 50% (M. Shiota, Vanderbilt Diabetes Center, unpublished data).

Importantly, in striatum—a region that contains abundant DATs [37–39] and IRs [15,17,18] and that participates in the reward pathway [1,2]—STZ treatment inhibited Akt activity. To assess Akt activity in these studies, we measured its ability to phosphorylate in vitro GSK3α [29]. As seen in Figure 1, STZ treatment reduces basal Akt activity, reflected by a decreased phosphorylation of GSK3α with respect to untreated controls. In three independent experiments, STZ treatment in rats led to a 44 ± 16% decrease in Akt activity measured from striatal synaptosomes (Figure 1B), suggesting that the STZ treatment significantly downregulates basal PI3K signaling in striatum.

**Figure 1:**
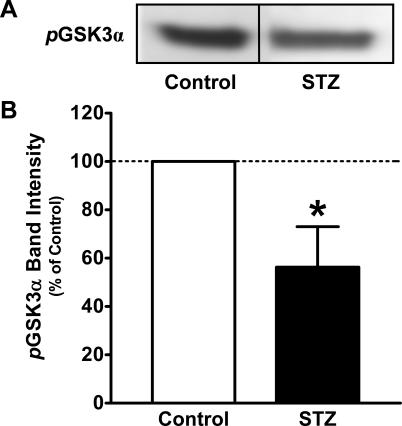
STZ Decreases Akt Activity in Rat Striatum (A) Representative immunoblots for phosphorylated GSK3α (pGSK3α), a downstream target of Akt, in either untreated subjects (control) or in those that received STZ 1 wk before sacrificing the animals. (B) Quantitation of pGSK3α immunoreactivity conducted in striatal synaptosomes 7 d after STZ (65 mg/kg, intravenous [i.v.]). The densities of the *p*GSK3α bands were normalized to their respective total protein concentrations (determined by protein assay; 100 μg) and then expressed as a percentage of control. *p < 0.05, paired Students *t*-test (*n* = 3).

To verify whether inhibition of PI3K signaling induced by STZ treatment correlates with changes in AMPH-induced DA efflux, we used HSCA to measure the release and clearance kinetics of striatal DA in vivo [14,40]. One week after STZ treatment (blood glucose: 495 ± 31 mg/dl [STZ-treated rats] versus 115 ± 5 mg/dl [saline-treated controls], *p* < 0.001, Students *t*-test; *n* = 6), HSCA recordings were carried out. In saline-treated rats, ejection of AMPH (400 μM/125 nl) caused a robust release of DA that was rapidly cleared from the extracellular space (Figure 2A). In contrast, AMPH elicited significantly less DA release in STZ-treated rats, and the released DA was cleared more slowly in these animals (Figure 2A). The slope of the rising portion of the DA signal indicates the rate of DAT-mediated DA efflux, which is primarily dependent on the affinity and turnover rate of DA and is independent of DA content [14,40]. Analysis of the rising phase of the trace revealed that DA efflux rates in STZ-treated rats were severely attenuated compared with those of saline-treated control rats (Figure 2B). STZ-treated rats also had a significantly lower amount of released DA (Figure 2C). Furthermore, STZ-treated animals also displayed significant deficits in DAT-mediated DA clearance, indicated by the reduced slope of the descending phase of the DA signal compared to saline-treated rats (Figure 2D). These data suggest that under hypoinsulinemic conditions, in which PI3K signaling is diminished, the ability of AMPH to cause DA efflux is impaired, possibly by decreasing DAT function.

**Figure 2:**
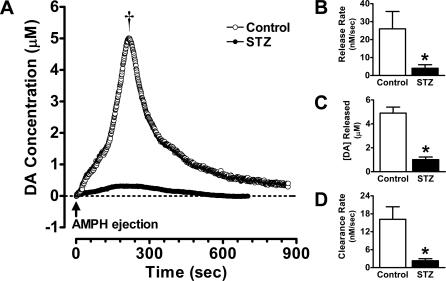
Depletion of Insulin Severely Blunts the Ability of AMPH to Release DA (A) Shown in the figure are integrated DA oxidation traces from a representative control rat and a STZ-treated rat, obtained using HSCA in the striatum. AMPH (400 μM/125 nl) was microinjected into the striatum (arrow), and extracellular DA levels were measured. (B–D) Summary data for HSCA, *p < 0.05, Students *t*-test for independent samples (*n* = 6). The slope of the rising portion of the DA signal—indicating the rate of AMPH-induced DA release—was decreased in STZ-treated rats compared to control (B), as was the amount of released DA (C). The slope of the descending phase of the DA signal, corresponding to the rate of clearance of AMPH-evoked DA, was also diminished in hypoinsulinemic rats (D).

### Pharmacological Inhibition of PI3K Signaling in Striatum Attenuates AMPH-Evoked DA Release

It is possible that factors other than PI3K signaling (e.g., altered blood glucose levels) might contribute to the blunted AMPH-induced DA release caused by STZ treatment. To address this concern, we selectively inhibited PI3K activity within the striatum of naive rats using LY294002 and then recorded AMPH-induced DA efflux in this region using HSCA. Figure 3 shows the effect of LY294002 pretreatment on AMPH-induced DA release. LY294002 (1 mM/125 nl) or vehicle (artificial cerebrospinal fluid [aCSF] in DMSO) were infused into the striatum by way of a calibrated micropipette positioned adjacent to the recording electrode. AMPH (400 μM/125 nl) was infused 0, 45 and 90 min later. The inhibition of PI3K led to a significant reduction in the ability of AMPH infusions to induce DA efflux 45 and 90 min after treatment (Figure 3).

**Figure 3:**
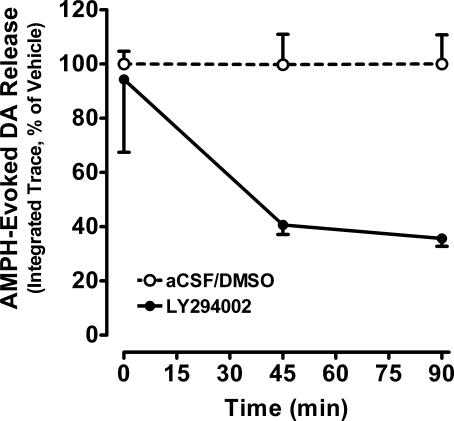
Inhibition of PI3K Decreases AMPH-Induced Striatal DA Release DA concentration was determined by digital integration of in vitro–calibrated oxidative currents using HSCA recordings in the striatum. The PI3K inhibitor LY294002 or vehicle (aCSF in DMSO) was microinjected into the striatum 300 μm away from the carbon fiber electrode. AMPH (400 μM/125 nl) was then ejected 0, 45 or 90 min later. Data represent peak AMPH-induced DA release expressed as a percentage of vehicle. Main effect treatment *p* < 0.005, two-way ANOVA; *p < 0.05, Bonferroni post hoc (*n* = 3–4).

The precise concentration of LY294002 or AMPH that reaches the recording site is unknown, because it depends on diffusion through the extracellular matrix [41]. However, it has been estimated that there is at least a 10-fold dilution in drug concentration when ejected from a micropipette at a distance of 300 μm from the recording electrode [42], which is the separation distance that was used in the current studies. Additional studies from our laboratory suggest that a 10- to 200-fold dilution in drug concentration occurs by the time it diffuses to the recording electrode [43]. Thus, a barrel concentration of 400 μM AMPH or 1 mM LY294002 when pressure-ejected into brain would yield concentrations at the recording electrode of approximately 2–40 μM or 5–100 μM, respectively. Previous studies have shown that these concentrations of AMPH are consistent with those reported in brain after systemic administration of a behaviorally effective dose of AMPH and its derivatives [44,45]. Furthermore, the concentrations of LY294002 are similar to those that are able to regulate cocaine sensitization [26].

### Hypoinsulinemia Reduces DAT Cell Surface Expression in Striatum

DAT is dynamically regulated at the plasma membrane by a number of intracellular signals [9,10,46,47], and recent data have also shown that transporter levels can be regulated by DA [48], pseudosubstrate stimulants such as AMPH [48,49], and inhibitors of DAT function such as cocaine [50]. To evaluate whether the reduction in AMPH-induced DA efflux caused by hypoinsulinemic conditions is promoted by a decrease in DAT cell surface expression, we evaluated DAT levels at the plasma membrane in striatal synaptosomes from rats made hypoinsulinemic with STZ [13]. As shown in Figure 4, chronic depletion of insulin results in a significant (>40%) decrease in the level of biotinylated, membrane-associated DAT within synaptosomes, indicating that DAT cell surface expression was significantly reduced in hypoinsulinemic rats. These findings, together with our electrochemical data (Figures 2 and 3), support the hypothesis that the reduction in AMPH-induced DA efflux caused by STZ treatment is a consequence of a reduction in DAT levels on the plasma membrane and are consistent with the previously reported blunted behavioral properties of AMPH under hypoinsulinemic conditions [12,33,34,51].

**Figure 4:**
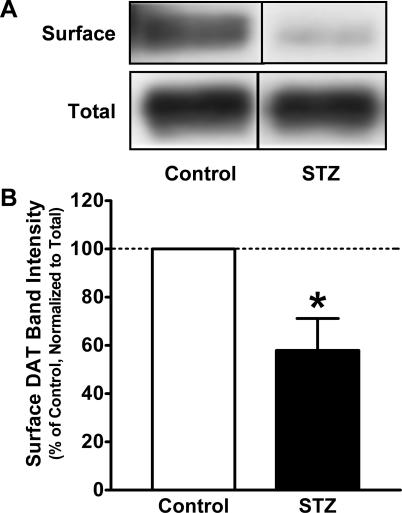
Cell Surface DAT Expression within Striatum Is Reduced in STZ-Treated Rats (A) Representative immunoblots for biotinylated (surface) and total DAT, in either untreated subjects (control) or in those that received streptozotocin (STZ). (B) Quantitation of DAT immunoreactivity from immunoblotting as in panel A conducted in striatal synaptosomes 7 d after STZ (65 mg/kg, i.v.). Biotinylated proteins (30 μg), representing 10% of the total lysate (300 μg protein) were separated by SDS-PAGE and underwent immunoblotting with a DAT-specific antibody; samples containing total lysates were run on adjacent lanes. Each biotinylated DAT band density was normalized to that of its corresponding total DAT band. Surface-to-total DAT ratios were then expressed as a percentage of control. *p < 0.05, Students *t*-test for independent samples (*n* = 3).

### Hypoinsulinemia Attenuates AMPH-Induced Blood Oxygenation Level–Dependent Responses in Striatum

To further explore noninvasively the effect of STZ, hypoinsulinemia and downregulation of the PI3K signaling on AMPH-induced DA efflux, we used blood oxygenation level–dependent (BOLD) fMRI, which is sensitive to fluctuations in blood/hemoglobin oxygenation that closely reflects changes in neuronal activity [52,53]. In recent years, fMRI has proven useful in the study of the neural and pharmacological properties of psychostimulants within small laboratory animals [54–60]. Notably, when examined in rodents, BOLD responses to AMPH are linearly correlated with AMPH-induced changes in extracellular DA levels within the striatum [54,55]. In the present study, the BOLD responsiveness of the DAT- and IR-rich striatum to AMPH stimulation in normal and hypoinsulinemic rats was measured at 9.4 T using T2*-weighted multi-slice gradient echo imaging. Figure 5 shows that STZ-pretreated rats displayed a marked reduction in striatal activation in response to an acute exposure to AMPH (3 mg/kg, intraperitoneal [i.p.]). Figure 5A depicts representative BOLD activation maps from untreated control versus STZ-treated animals, each co-registered to high-resolution anatomic templates acquired in the same animals. Compared to untreated control rats given acute saline, those receiving AMPH exhibited significant BOLD activation in the dorsolateral striatum. However, this response was absent in rats rendered hypoinsulinemic by STZ treatment.

**Figure 5:**
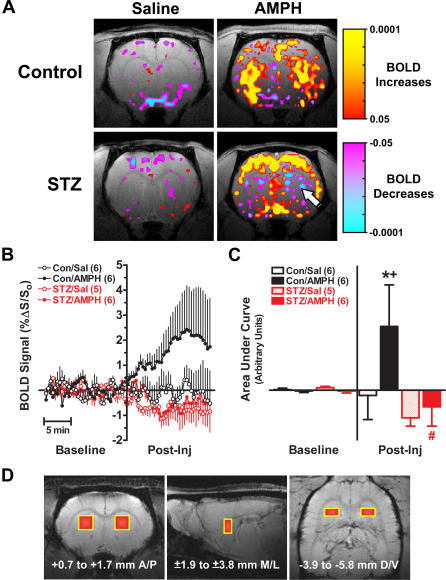
AMPH-Induced Striatal BOLD fMRI Responses Are Attenuated in STZ-Treated Rats (A) *t*-thresholded statistical maps (*p* < ±0.05 to *p* < ±0.0001; uncorrected comparisons of 15-min baseline period versus 15-min post-injection period) were constructed from subjects that best represent the BOLD signal activation within the DAT- and IR-enriched dorsal striatum. BOLD signal changes in response to saline (left panels) versus acute AMPH (right panels) within untreated control rats (top panels) versus STZ-treated rats (bottom panels). Compared to drug-naive animals given saline, AMPH [3 mg/kg, i.p.] elicited considerable BOLD activation in the dorsal striatum. This response was absent in drug-naive, STZ-pretreated hypoinsulinemic rats (arrow). (B) ROI analyses of striatal BOLD fMRI data for all subjects individually for each of the four treatment conditions in Panel A (*n* = 5–6). (C) Integration of fMRI time series data reveals significant group differences in BOLD activation within the dorsal striatal ROIs. One-way ANOVA (*F*_7,56_ = 2.83, *p* < 0.05); Newman-Keuls (*p* < 0.05 compared to Baseline*, Saline+ and Control#). (D) Three-dimensional stereotaxic orientation of the striatal ROIs (1.88 × 1.88 × 1 mm) analyzed in (B and C). Shown at the bottom of each slice—representing axial, sagittal and coronal orientations—are the corresponding stereotaxic coordinates for the ROIs [68].

To quantify the effects of hypoinsulinemia on the striatal BOLD signal we performed region-of-interest (ROI) analysis of dorsolateral portions of this structure, which is predominantly innervated by the substantia nigra compacta and where *t*-maps indicated strong AMPH-evoked BOLD activation that was sensitive to insulin depletion. Figure 5B–5D summarizes the results of this analysis across groups of subjects (*n* = 5–6 per treatment group). When compared to treatment with saline, AMPH-treated animals exhibited a strong BOLD signal increase above baseline in the dorsolateral striatum. In contrast, there was no significant AMPH-induced BOLD signal change from baseline in STZ-pretreated, insulin-depleted rats (Figure 5B). In Figure 5C, post-injection traces from animals within each of the four treatment conditions described in Figure 5A and 5B were integrated and compared using one-way analysis of variance (ANOVA): *F*_3,31_ = 3.30, *p* < 0.05. Multiple comparisons between group pairs were conducted post hoc using the Newman-Keuls test: *p* < 0.05 compared to ^*^Baseline, ^+^Saline and ^#^Untreated Control. ROI analysis of the ventral striatum (nucleus accumbens, NAc), which is innervated by the ventral tegmental area (VTA), revealed that the BOLD response to AMPH challenge did not significantly differ between STZ-treated and control animals (unpublished data). Likewise, prelimbic and cingulate cortices, also innervated by the VTA and where the norepinephrine transporter is the predominant carrier supporting DA inactivation [61], failed to show significant differential AMPH-induced BOLD responses after STZ (unpublished data).

### Intrastriatal Insulin Infusion Restores AMPH Action and DAT Function in STZ-Treated Animals

To further elucidate the links between the PI3K signaling pathway, DAT function and AMPH action, we activated the PI3K pathway pharmacologically within the striatum of STZ-treated, hypoinsulinemic animals by locally infusing insulin just before the delivery of a brief AMPH pulse in this region. One week after depleting insulin with STZ treatment, local (striatal) application of exogenous insulin (10μM/100 nl) 2 min before AMPH infusion (400 μM/125 nl) almost fully restored to control levels the rate and the amount of AMPH-evoked DA release (Figure 6A and 6B), as well as the rate of DAT-mediated clearance (Figure 6C). These data further support our hypothesis that PI3K signaling is crucial for AMPH to stimulate DA efflux.

**Figure 6:**
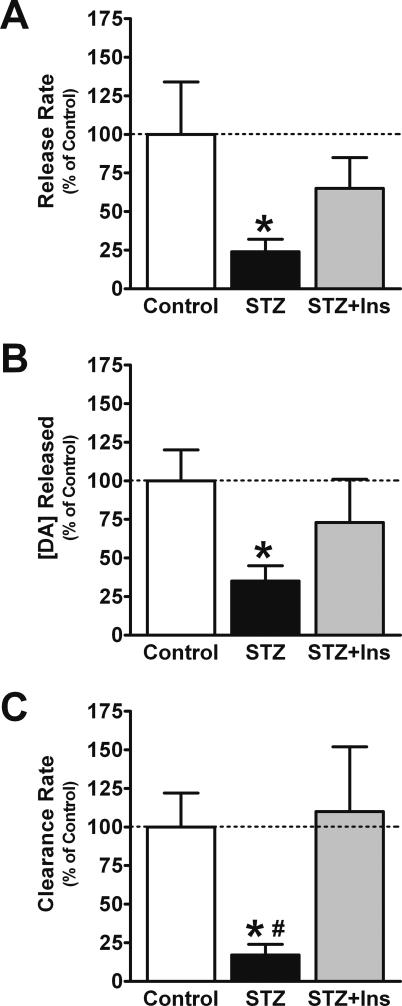
Local Infusion of Insulin Restores AMPH-Evoked DA Release in Striatum of STZ-Treated Rats Summary data for HSCA (*n* = 5–7). When measured in the dorsal striatum and compared to saline-treated control subjects, STZ-induced depletion of insulin (STZ) resulted in a significant reduction in the rate of DA efflux in response to local injection of AMPH (400 μM/125 nl; (A) as well as a decrease in the maximum amount of released DA (B) and the rate of DA clearance (C) with respect to control. These findings provide a replication of those shown in Figure 2 in a separate cohort of rats. In contrast, when exogenous insulin (10 μM/100 nl) is locally applied in STZ animals 2 min prior to AMPH (STZ+Ins), all three of these electrochemical parameters are normalized to levels approximating control. One-way ANOVA (*F*_2,14_ = 2.46; *p* < 0.05); Mann-Whitney (*p* < 0.05 compared to Control* and STZ+Ins#).

## Discussion

In recent years, the PI3K/Akt signaling pathway has been heavily implicated in the development, progression and maintenance of drug dependence [26–28]. Regulation of DAT plasma membrane expression (and subsequently of extracellular DA) by PI3K signaling is emerging as an important mechanism linking neurotransmitter transporter function to psychomotor stimulant abuse [11,13]. Profound adaptations within the neuronal dopaminergic system occur in experimentally induced diabetic mice [51]. Compared to controls, STZ-treated hypoinsulinemic rats display a marked reduction in striatal DA clearance [14,30] and are resistant to the behavioral effects of AMPH [12,33,34,51]. In experimentally induced diabetic rats (i.e., alloxan-treated), AMPH administered acutely is less effective at producing anorexia and stereotyped behavior and at increasing locomotor activity; subsequent administration of insulin reverses this attenuated sensitivity to AMPH [33]. Importantly, Galici et al. [12] showed that there is a selective decrease in AMPH self-administration in diabetic rats, consistent with data showing that dopamine uptake is decreased in hypoinsulinemic rats [14,30]. Considering that the striatum is highly enriched in insulin [17,62] and IRs [15,17,18], as well as in DAT [37–39], these studies strongly support a role for the neuronal PI3K pathway in regulating DAT activity and extracellular DA levels, as well as in the actions of AMPH.

The link between the PI3K pathway and the actions of AMPH is further fortified by recent studies from our laboratory as well as others, showing that prolonged exposure to AMPH ex vivo and in vivo inhibits PI3K signaling, as measured by Akt activity in striatum [29,63]. Akt is a protein kinase that is immediately downstream of PI3K, and Akt activity has been shown to be essential for insulin modulation of transporter function in striatal synaptosomes and human DAT-expressing cells [11,13]. Indeed, insulin signaling increases DA uptake capacity and cell surface expression [11,13]. In contrast, in vitro inhibition of either PI3K or Akt causes a decrease in DA uptake capacity and a redistribution of DAT away from the plasma membrane [11,13].

Here we demonstrate in vivo that hypoinsulinemia and pharmacological inhibition of PI3K signaling reduces the ability of AMPH to evoke DA efflux in the striatum. The reduction in DA efflux determined by HSCA in the current studies may result either from a decreased DAT plasma membrane expression, as suggested by in vitro [11,13] and ex vivo [14,30] studies, from a diminished DA content [64], or from both. Our data suggest that it is unlikely that the reduced DA efflux is a consequence of changes in tissue DA content. This is because the analysis of the rising phase of the HSCA traces revealed that the rate of AMPH-induced DA efflux in STZ-treated rats was severely attenuated compared with that of saline-treated control rats (Figure 2B). In fact, the slope of the rising portion of the DA signal represents the rate of DAT-mediated DA efflux, which is primarily dependent on the affinity and turnover rate of DA and is independent of DA content [14,40]. In addition, DA clearance as measured by HSCA that is independent of DA content but dependent on DAT number at the plasma membrane is reduced in STZ-treated animals [14]. STZ treatment does not significantly influence total DAT number or DA affinity [12,14]. Thus, the current findings suggest that a reduction in insulin signaling leads to a decrease in DAT function, a notion supported by the previous study from Owens et al. [14] showing that AMPH-naive, STZ-treated rats exhibit significantly less DA uptake in striatum as determined in vivo with HSCA and ex vivo in synaptosomes. Collectively these data support the hypothesis that hypoinsulinemia, by downregulation of PI3K signaling (see Figure 3), significantly reduces AMPH-induced DA efflux because of reduced DAT plasma membrane expression.

In support of the current in vivo electrochemical and ex vivo biochemical findings, STZ treatment was also found to inhibit the ability of AMPH to induce a BOLD response in the dorsal striatum. The current study did not reveal significant differences in insulin-dependent, AMPH-induced BOLD signal fluctuations in the NAc (unpublished data). Possibly, this was due to the limited radio frequency penetration of the surface coil used in this study into more ventral brain areas such as the NAc. Importantly, others have reported decreases in AMPH-induced DA release in NAc dialysates collected in freely moving rats [65]. Because hyperglycemia has been shown to not significantly influence BOLD signals [66], our data suggest that blunting of the AMPH-induced BOLD response in the DAT-dense striatum of insulin-depleted rats (Figure 5) is not due to STZ-mediated metabolic abnormalities. Importantly, in the striatum, the AMPH-induced BOLD response has been shown to correlate with striatal extracellular DA levels [54,55] and, consequently, with DAT-mediated reverse transport of DA. These data further support our hypothesis that STZ treatment, by decreasing PI3K signaling in striatum, downregulates AMPH-induced DA efflux measured by HSCA (Figures 2, 3 and 6) and fMRI (Figure 5). Our results are consistent with in vitro studies demonstrating that the blockade of insulin signaling decreases the number of active DATs on the plasma membrane [13] as we currently demonstrate in DAT cell surface biotinylation studies from striatal preparations (Figure 4). These data support the hypothesis that the attenuated rate of AMPH-induced striatal DA efflux in hypoinsulinemic rats results from a DAT trafficking phenomenon [14]. Conceivably, in STZ-treated animals, insulin stimulation of PI3K signaling should restore DA clearance and AMPH-induced DA efflux. Figure 6 shows that local application of insulin in STZ-treated rats almost completely restores AMPH-stimulated DA efflux.

We demonstrate here that PI3K signaling regulates the pharmacological actions of drugs (e.g., AMPH) that act on dopaminergic systems. Importantly, our data show that hypoinsulinemia reduces basal PI3K signaling and impairs the ability of AMPH to increase extracellular DA levels. Therefore, PI3K signaling may provide a new cellular target for the development of novel treatments of AMPH abuse and regulation of dopaminergic tone.

## Materials and Methods

### STZ treatments.

All procedures were approved by the Vanderbilt University Medical Center and the University of Texas Health Science Center at San Antonio Institutional Animal Care and Use Committees and were conducted according to the National Institutes of Health Guide for the Care and Use of Laboratory Animals. For all experiments, male Sprague–Dawley (Harlan, Indianapolis, Indiana, United States) rats (275–350 g) served as experimental subjects. STZ is an antibiotic that destroys the insulin-secreting β cells of the pancreas [36] and has previously been used to induce chronic hypoinsulinemia in rats by our laboratories [12,14]. STZ (Sigma-Aldrich; http://www.sigmaaldrich.com) was freshly dissolved in ice-cold 100 mM citrate saline (pH 4.5) for all studies. Rats received STZ (50 mg/kg, i.p. for HSCA studies; 65 mg/kg into the tail vein for fMRI studies) and were returned to their home cages for at least 7 d. Blood glucose was measured with a glucometer (Advantage Accu-Chek, Roche Diagnostics; http://www.roche.com) before STZ and just before an experiment. Animals were considered hypoinsulinemic when their glucose levels exceeded 300 mg/dl.

### Synaptosome preparation.

Preparation of synaptosomes was performed as described previously [11,12,14]. Rats were killed by decapitation, their brains were removed and their striata were rapidly dissected on a plastic dish placed on ice. Tissue was homogenized in ice-cold Krebs-Ringer buffer (125 mM NaCl, 1.2 mM KCl, 1.2 mM MgSO_4_, 1.2 mM CaCl_2_, 22 mM NaHCO_3_, 1 mM NaH_2_PO_4_, 10 mM glucose, pH 7.4) containing 0.32 M sucrose using a glass-Teflon homogenizer. Homogenates were centrifuged at 1,000*g* for 10 min at 4 °C, and the resulting supernatants were centrifuged at 16,000*g* for 25 min at 4 °C. P2 pellets were then placed on ice and resuspended immediately prior to experiments.

### Assay of Akt activity.

Akt activity assays were performed as described previously [29]. Striatal synaptosomes were lysed for 45 min at 4°C in a buffer containing 20mM Tris (pH 7.5), 150 mM NaCl, 1 mM EDTA, 1 mM EGTA, 1% Triton X-100, 2.5 mM sodium pyrophosphate, 1 mM β-glycerolphosphate, 1 mM Na_3_VO_4_, 1 μg/ml leupeptin and 1 mM PMSF. Lysed proteins (∼400 μg; BioRad DC Protein Assay Kit; http://www.biorad.com) underwent immunoprecipitation with an Akt-specific monoclonal antibody as part of a commercially available Akt activity assay kit (BioVision; http://www.biovision.com). Activity of the immunoprecipitated Akt was determined in vitro with the addition of recombinant GSK3α as the kinase substrate; the resulting phosphorylated GSK3α (pGSK3α) was determined by immunoblotting (see below) using phosphospecific antibodies to GSK3α (Ser 21, diluted 1:1000), provided in the Akt activity assay kit.

### Biotinylation of cell surface DAT.

Biotinylation studies were performed as described previously [13,48,49] with modification. Striatal synaptosomes were washed twice with warm Krebs–Ringer bicarbonate (KRB) buffer (containing 145 mM NaCl, 2.7 mM KCl, 1.2 mM KH_2_PO_4_, 1 .2 mM CaCl_2_, 1.0 mM MgCl_2_, 10 mM glucose, 0.255 mM ascorbic acid, and 24.9 mM NaHCO_3_) and then incubated in the same buffer for 1 h at 37 °C. The reaction was stopped in ice and the samples were washed with phosphate-buffered saline ( PBS) containing 0.1 mM CaCl_2_ and 1 mM MgCl_2_ (PBS-Ca-Mg) and incubated with EzLink Sulfo-NHS-SS-Biotin (2.0 mg/ml in PBS-Ca-Mg; Pierce Chemical; http://www.piercenet.com) on ice for 30 min. The reaction was quenched by washing twice with 4 °C PBS-Ca-Mg containing 100 mM glycine (PBS-Ca-Mg-glycine) followed by an incubation with PBS-Ca-Mg-glycine for 15 min on ice. Synaptosomes were then washed twice with cold PBS-Ca-Mg before lysis with 1 ml of radioimmunoprecipitation assay (RIPAE) buffer (10 mM Tris pH 7.4, 150 mM NaCl, 1 mM EDTA, 0.1% SDS, 1% sodium deoxycholate and 1% Triton X-100) containing protease inhibitors (0.5 mM phenylmethylsulfonyl fluoride, 5 μg/ml leupeptin and 5 μg/ml pepstatin) for 1 h 30 min on ice with agitation. Lysates were then centrifuged at 14,000*g* for 30 min at 4 °C. After isolation of supernatants, biotinylated proteins were separated by incubation with 90 μl ImmunoPure immobilized streptavidin beads (Pierce) for 1 h at room temperature with agitation. Beads were washed three times with RIPAE buffer; biotinylated proteins were then eluted in 50 μl of 2X SDS-PAGE sample loading buffer at room temperature. Total cell lysates (∼300 μg protein) and the biotinylated (cell surface) fraction (∼10% of total; 30 μg protein) underwent immunodetection for DAT as described below.

### Immunoblot detection of DAT.

Determination of biotinylated DAT immunoreactivity was conducted with some modification according to previously described methods [13,29]. Briefly, synaptosomal lysates were separated by SDS-PAGE, and resolved proteins were transferred to polyvinylidene difluoride (PVDF) membranes (BioRad), which were incubated for 1–2 h in blocking buffer (5% dry milk and 0.1% Tween 20 in Tris-buffered saline). To quantify biotinylated (surface) DAT, immunoblots were incubated with mouse monoclonal primary antibodies to the N terminus of the rat DAT (antibody 16, 1:1000, [67]), generously provided by Roxanne Vaughan (University of North Dakota School of Medicine and Health Sciences, Grand Forks, North Dakota, United States). All proteins were detected using HRP-conjugated goat anti-mouse secondary antibodies (1:5000; Santa Cruz Biotechnology; http://www.scbt.com). After chemiluminescent visualization (ECL-Plus; Amersham; http://www.amersham.com) on Hyperfilm ECL film (Amersham), protein band densities were quantified (Scion Image; http://www.scioncorp.com) and normalized to the appropriate total protein amount. Immunoblotting experiments were performed in triplicate, analyzed (GraphPad v4.0; http://www.graphpad.com) and reported as mean ± standard error of the mean.

### HSCA.

HSCA was conducted using the FAST-12 system (Quanteon; http://www.quanteon.cc) as previously described with some modification [14]. Recording electrode/micropipette assemblies were constructed using a single carbon-fiber (30 μm diameter; Specialty Materials; http://www.specmaterials.com), which was sealed inside fused silica tubing (Schott, North America; http://www.schott.com). The exposed tip of the carbon fiber (150 μm in length) was coated with 5% Nafion (Aldrich Chemical Co.; http://www.sigmaaldrich.com; 3–4 coats baked at 200 °C for 5 min per coat) to provide a 1000-fold selectivity of DA over its metabolite dihydroxyphenylacetic acid (DOPAC). Under these conditions, microelectrodes displayed linear amperometric responses to 0.5–10 μM DA during in vitro calibration in 100 mM phosphate-buffered saline (pH 7.4).

Animals were anesthetized with injections of urethane (850 mg/kg, i.p.) and α-chloralose (85 mg/kg, i.p.), fitted with an endotracheal tube to facilitate breathing, and placed into a stereotaxic frame (David Kopf Instruments; http://www.kopfinstruments.com). To locally deliver test compounds (see below) close to the recording site, a glass single or multi-barrel micropipette (FHC; http://www.fh-co.com) was positioned adjacent to the microelectrode using sticky wax (Moyco; http://www.moycotech.com). The center-to-center distance between the microelectrode and the micropipette ejector was 300 μm. For experiments in Figure 2, the micropipette was filled with AMPH (400 μM; Sigma) or its vehicle (PBS). The study in Figure 3 used a multibarrel configuration in which barrels contained AMPH (400 μM) or vehicle (aCSF) and additional barrels contained LY294002 (1 mM; Sigma) or its vehicle (aCSF in DMSO). For experiments in Figure 6, one barrel contained AMPH (400 μM) and an adjacent barrel contained insulin (10 μM; Sigma); a third barrel contained aCSF, the vehicle for both AMPH and insulin. The electrode/micropipette assembly was lowered into the striatum at the following coordinates (in mm from bregma [68]): A/P, +1.5; M/L, ±2.2; D/V, −3.5 to −5.5. The application of drug solutions was accomplished using a Picospritzer II (General Valve Corporation; http://www.parker.com) in an ejection volume of 100–150 nl (5–25 psi for 0.25–3 s). After ejection of test agents, there is an estimated 10–200-fold dilution caused by diffusion through the extracellular matrix to reach a concentration of 2–40 μM (AMPH), 5–100 μM (LY294002) or 0.05–1 μM (insulin) at the recording electrode [43]. To record the efflux and clearance of DA at the active electrode, oxidation potentials—consisting of 100-ms pulses of 550 mV, each separated by a 1-s interval during which the resting potential was maintained at 0 mV—were applied with respect to an Ag/AgCl reference electrode implanted into the contralateral superficial cortex. Oxidation and reduction currents were digitally integrated during the last 80 ms of each 100-ms voltage pulse. For each recording session, DA was identified by its reduction/oxidation current ratio: 0.55–0.80.

At the conclusion of each experiment, an electrolytic lesion was made to mark the placement of the recording electrode tip. Rats were then decapitated while still anesthetized, and their brains were removed, frozen on dry ice, and stored at −80°C until sectioned (20 μm) for histological verification of electrode location within the striatum. HSCA data were analyzed with GraphPad Prism using three signal parameters (see Figure 2A for exemplary trace): (i) the DA efflux rate (in nM/s), which is the change in DA oxidation current evoked by AMPH application as a function of time; (ii) the maximal signal amplitude of the released DA (in μM); and (iii) the DA clearance rate (in nM/s), defined as the slope of the linear portion of the current decay curve, i.e., from 20−60% of maximal signal amplitude.

### fMRI.

Under isoflurane anesthesia, rats were implanted with femoral artery and i.p. catheters, tracheotomized and artificially ventilated with a 30:70% O_2_:N_2_O mixture. Rats were paralyzed with a bolus infusion of pancuronium bromide (2 mg/kg; Sigma) dissolved in isotonic saline (1 ml/kg, i.p.), and the concentration of isoflurane was reduced to 0.88%. Ventilation parameters were adjusted (respiratory rate = 48–52 breaths/min; inspiration volume 14–18 cm H_2_O) to maintain stable blood gases, which were sampled from the arterial catheter immediately before and after the completion of MRI scans. Mean arterial gas values obtained from all 23 rats used in fMRI studies were: pH = 7.36 ± 0.06, pCO_2_ = 37.7 ± 6.8 mm Hg, pO_2_ = 140.5 ± 20.6 mm Hg. A respiration pillow sensor (SA Instruments; http://www.i4sa.com) was positioned underneath the animal's abdomen. Core body temperature and heart rate were monitored during imaging studies using a rectal probe and subdermal electrocardiograph (ECG) electrodes implanted into the forepaws. Temperature, ECG and respiratory data were collected and analyzed using an MR-compatible monitoring system (SAM-PC, SA Instruments).

To minimize motion artifacts, rats were positioned within a custom-built plexiglass stereotaxic platform and fixed in place with Teflon ear bars and an adjustable incisor bar. Attached to the platform was a socket holding a 20-mm dual transmit-receive radio frequency surface coil (Varian Instruments; http://www.varianinc.com) lowered to 1 mm above the scalp. The platform was then placed inside a 9.4 T, 21-cm bore Varian Inova superconducting magnet equipped with actively shielded gradients of 40 G/cm and peak rise times of 135 μs. The MRI system was controlled by a Varian console interfaced with a Sun Microsystems computer running VnmrJ 6.1D software (Varian). Nine contiguous coronal slices, serving as within-subject high-resolution anatomic templates, were acquired using conventional gradient echo multi-slice (GEMS) imaging. Seventy-two functional image volumes, spatially aligned with the anatomic templates, were then continuously acquired for 30 min using the following GEMS parameters: TR/TE = 220/12 ms; flip angle = 20°; NEX = 2; slice thickness = 1 mm; in-plane voxel resolution = 0.47 × 0.47 mm; matrix = 64 × 64; FOV = 30 × 30 mm; acquisition time = 25.6 s per excitation. After a 15-min baseline period, AMPH dissolved in isotonic saline was administered as a bolus i.p. infusion (3 mg/ml/kg; 20–30 s); image acquisition continued uninterrupted for 15 min after the infusion.

Analysis of fMRI data was conducted in MATLAB (v7.0.4; The MathWorks; http://www.mathworks.com) as described previously, with some modification [69]. Data were first analyzed to generate statistical parametric activation maps based on the Student's *t*-test. *t* values were computed for each image voxel by comparing the baseline signal to the post-injection signal. For each subject, colorized *t* values from each image voxel were registered to a high-resolution anatomic template obtained in the same subject. ROI analyses were conducted over the dorsolateral striatum, the ventral striatum (NAc) and prelimbic/cingulate cortices based on 1-mm-thick coronal slices spanning 1–2 mm anterior to bregma [68]. For each ROI, fMRI time series data underwent baseline drift correction with a linear detrending function; high-frequency noise was suppressed with a low-pass Butterworth filter. Pixel intensities from each image voxel in the ROIs were converted to percent signal changes from baseline (% ΔS/So), averaged across left and right hemispheres, integrated and analyzed by one-way ANOVA followed by a Newman-Keuls post test.

## Abbreviations

aCSF: artificial cerebrospinal fluid
AMPH: amphetamine
ANOVA: analysis of variance
BOLD: blood oxygenation level–dependent
DA: dopamine
DAT: dopamine transporter
fMRI: functional magnetic resonance imaging
HSCA: high-speed chronoamperometry
IGF: insulin-like growth factor
i.p.: intraperitoneal
IR: insulin receptor
NAc: nucleus accumbens
PI3K: phosphotidylinositol 3-kinase
ROI: region of interest
RTK: receptor tyrosine kinase
STZ: streptozotocin
